# Supplementary material for: Targeting a future generation free from female genital mutilation: A mixed-methods quasi-experimental study of an awareness intervention in central Tanzania
Source: PLOS Glob Public Health. 2026 May 26;6(5):e0006365. doi: 10.1371/journal.pgph.0006365 (PMC13210218; doi:10.1371/journal.pgph.0006365)
Supplement: S2 Checklist — (PDF) [file pgph.0006365.s002.pdf]

S2 Checklist. Consolidated criteria for reporting qualitative studies (COREQ): 32-item checklist

Manuscript: Targeting a future generation free from female genital mutilation: a mixed-methods quasi-experimental study of an awareness intervention in central Tanzania

**No. Item**	**Guide questions/description**	**Reported on Page #**
**Domain 1: Research team and reflexivity**		
Personal Characteristics		
1. Interviewer/facilitator	Which author/s conducted the interview or focus group?	**MNI & LBK:**<br>Data collection, page 13-14
2. Credentials	What were the researcher's credentials? E.g., PhD, MD	Authors' affiliation & Information, page 1
3. Occupation	What was their occupation at the time of the study?	Authors' affiliation & Information, page
4. Gender	Was the researcher male or female?	Both sexes:<br>Authors' affiliation & Information, page 1
5. Experience and training	What experience or training did the researcher have?	Authors' affiliation & Information, page 1
Relationship with participants		
6. Relationship established	Was a relationship established prior to study commencement?	**Yes:**<br>Study design and registration, page 4<br>Interventions, page 10-11<br>Ethical considerations, page 35
7. Participant's knowledge of the interviewer	What did the participants know about the researcher? e.g., personal goals, reasons for doing the research	Interventions, page 10-11<br>Data collection, page 10-11<br>Ethical considerations, page 35
8. Interviewer characteristics	What characteristics were reported about the interviewer/facilitator? e.g., Bias, assumptions, reasons, and interests in the research topic	Data collection, page 10-11, final paragraph

**Domain 2: study design**		
Theoretical framework		
9. Methodological orientation and Theory	What methodological orientation was stated to underpin the study?	**Phenomenology:**<br>Participants' sampling, recruitment,

	e.g. grounded theory, discourse analysis, ethnography, phenomenology, content analysis	and eligibility criteria, page 7-8, fourth paragraph
Participant selection		
10. Sampling	How were participants selected? e.g. purposive, convenience, consecutive, snowball	**Purposive:**<br>Participants' sampling, recruitment, and eligibility criteria, page 7-8, fourth paragraph
11. Method of approach	How were participants approached?<br>e.g. face-to-face, telephone, mail, email	**Face-to-face:**<br>Data collection, page 10-11, final paragraph
12. Sample size	How many participants were in the study?	**10 young mothers from hospitals**<br>Abstract page 2<br>Participants' sampling, recruitment, and eligibility criteria, page 7-8<br>Results- Qualitative findings: lived experiences, systemic barriers, and participant-driven solutions, first paragraph

13. Non-participation	How many people refused to participate or dropped out?<br>Reasons?	16 losses to follow up (baseline-endline survey): Attrition analysis, 18 and Table 4<br><br>72 excluded in clinical audit (missing consent: n=48); refusal: n=24: Participants' sampling,<br>recruitment, and eligibility criteria, page 7-8, final paragraph<br><br>Household survey (0): Interventions (10-11), final paragraph
		Four declined in qualitative interviews: Participants' sampling, recruitment, and eligibility criteria, page 7-8, fourth paragraph
Setting		
14. Setting of data collection	Where was the data collected? e.g., home, clinic, workplace	Hospitals and schools: Study setting and participants, page 5-7;<br>Participants' sampling, recruitment, and eligibility criteria, page 7-8; Table 2: Participants' demographics, page 17
15. Presence of non-participants	Was anyone else present besides the participants and researchers?	**No:**<br>Data collection, page 10-11, final paragraph

16. Description of sample	What are the important characteristics of the sample? e.g., demographic data, date	Table 2, page 17 (for baseline-endline characteristics)<br>Table 5, page 23 (for clinical audit)<br>Qualitative findings: lived experiences, systemic barriers, and participant-driven solutions, first paragraph, page 23 (for qualitative sample)
Data collection		
17. Interview guide	Were questions, prompts, guides provided by the authors? Was it pilot tested?	**Yes:**<br>Data collection, page 10-11; Results (16-27); S4 File
18. Repeat interviews	Were repeat interviews carried out? If yes, how many?	**No:**<br>Data collection, page 10-11, final paragraph
19. Audio/visual recording	Did the research use audio or visual recording to collect the data?	**Audio-recording:**<br>Data collection, page 10-11, final paragraph
20. Field notes	Were field notes made during and/or after the interview or focus group?	**Yes:**<br>Data collection, page 10-11, final paragraph
21. Duration	What was the duration of the interviews or focus group?	**30–60 minutes:**<br>Data collection, page 10-11, final paragraph

22. Data saturation	Was data saturation discussed?	**Yes:**<br>Participants' sampling, recruitment, and eligibility criteria, page 7-8, fourth paragraph
23. Transcripts returned	Were transcripts returned to participants for comment and/or correction?	**Not returned:**<br>Data collection, page 10-11, final paragraph
**Domain 3: analysis and findings**		
Data analysis		
24. Number of data coders	How many data coders coded the data?	**Two coders and validation by the entire team:**<br>Data analysis, page 14-15, final paragraph
25. Description of the coding tree	Did authors provide a description of the coding tree?	**Yes:**<br>Braun & Clarke six steps thematic analysis: Data analysis, page 14-15, final paragraph
26. Derivation of themes	Were themes identified in advance or derived from the data?	**Inductively derived:**<br>Data analysis, page 14-15, final
		paragraph
27. Software	What software, if applicable, was used to manage the data?	Manual coding: Data analysis, page 14-15, final paragraph
28. Participant checking	Did participants provide feedback on the findings?	**N/A**<br>Only member checking at the end of each interview was done for feasibility: Data collection, page 10-11, final paragraph

Reporting		
29. Quotations presented	Were participant quotations presented to illustrate the themes/findings? Was each quotation identified? e.g. participant number	**Yes:** Results - Qualitative Findings: Lived Experiences, Systemic Barriers, and Participant-Driven Solutions), page 23-27
30. Data and findings consistent	Was there consistency between the data presented and the findings?	**Yes:** Results - Qualitative Findings: Lived Experiences, Systemic Barriers, and Participant-Driven Solutions), page 23-27
31. Clarity of major themes	Were major themes clearly presented in the findings?	**Yes:** Results - Qualitative Findings: Lived Experiences, Systemic Barriers, and Participant-Driven Solutions), page 23-27
32. Clarity of minor themes	Is there a description of diverse cases or discussion of minor themes?	**Yes:** Results - Qualitative Findings: Lived Experiences, Systemic Barriers, and Participant-Driven Solutions), page 23-27

Reference Tong A, Sainsbury P, Craig J. Consolidated criteria for reporting qualitative research (COREQ): a 32item checklist for interviews and focus groups. Int J Qual Health Care. 2007 Dec;19(6):349-57. doi: 10.1093/intqhc/mzm042. Epub 2007 Sep 14. PMID: 17872937.
