# Supplementary material for: Targeting a future generation free from female genital mutilation: A mixed-methods quasi-experimental study of an awareness intervention in central Tanzania
Source: PLOS Glob Public Health. 2026 May 26;6(5):e0006365. doi: 10.1371/journal.pgph.0006365 (PMC13210218; doi:10.1371/journal.pgph.0006365)
Supplement: S1 Data — (PDF) [file pgph.0006365.s004.pdf]

S1 Data: Data Collection Instruments

Manuscript: Targeting a future generation free from female genital mutilation: a mixed-methods quasi-experimental study of an awareness intervention in central Tanzania

**INSTRUMENT 1:**	**Knowledge, Attitudes, and Perceptions on Female Genital Mutilation (FGM) Structured Questionnaire (English Version)**
**Participant Code Number**	**Fill your assigned number from (P1 to P468)**
**Instructions**	**Tick (v) or fill where applicable**

PART A: Participants' Demographics

SN	PARTICULAR		DESCRIPTION (If any)
1	Your age (in years)		
2	Sex		**[ ]** Male **[ ]** Female
3	Your ward of residence (mention name)		
4	Type of ward		[ ] Semi-urban [ ] Remote
5	Setting	School	Name:
		Hospital	Name:
6	Years of community residence		
7	Education level		[ ] College/University<br>[ ] Secondary<br>[ ] Primary<br>[ ] Not attended
8	Your tribe		

PART B: Awareness of Female Genital Mutilation Health Risks
Scoring: 1 mark per each correct response

	**Total correct points contribution in the awareness score per item**

1	FGM is the partial or total removal of external female genitalia and infliction of other injuries to female genitalia for non-medical reasons	[ ] Yes [ ] No	1
2	Source of information and awareness regarding FGM?<br>(tick all that apply)	[ ] Community discussions<br>[ ] School education<br>[ ] Mass media<br>[ ] Witness within family, relatives or neighbors	Excluded: Analysed separately
		[ ] FGM positive status (women only)<br>[ ] Previous involved in any anti-FGM program<br>Others (specify)…………………………	
3	Is FGM harmful?	**[ ] Yes [ ] No**	**1**
4	If yes in question 3 above, what could be the side effects of FGM?<br>(Tick (v) all that apply)	[ ] Infections<br>[ ] Excessive bleeding<br>[ ] Severe pain<br>[ ] Difficult childbirth<br>[ ] Urinary problems<br>[ ] Sexual problems<br>[ ] Psychological problems<br>Others (specify)……………………	**7**
5	Is FGM still occurring in the community?	[ ] Yes [ ] No	Excluded: Analysed separately
6	If yes in question 5 above, what could be the associated sustaining reasons?		Excluded: Analysed separately
7	Does FGM violate the human rights of girls and women?	[ ] Yes [ ] No	1

8	If yes in question 7 above, what could be the human rights violation associated with FGM?<br>(Tick (v) all that apply)	[ ] Intimate partner violence [ ] Child, early, and forced marriage<br>[ ] Stigma and gender discrimination<br>[ ] Right to health, security, and physical integrity<br>[ ] Right to life in cases of death [ ] Right to be free from torture and cruel, inhuman or other degrading treatment<br>Others (specify)…………………….<br>□	**6**
9	Is FGM a criminal offense in Tanzania and international laws?	[ ] Yes [ ] No	1
10	Are you aware of the ‘International Day of Zero Tolerance for FGM, February 6’, and its aim?	[ ] Yes [ ] No	1
11	Do you wish for FGM abandonment?	[ ] Yes [ ] No	1
12	Do you feel this project belongs to you/your community? (endline)□□	[ ] Yes [ ] No	Excluded: Analysed separately
□Excluded from awareness level estimation and paired analysis; □Explored only at baseline (no need for comparison).			

**INSTRUMENT 2**	**PART B: Chamwino District FGM Prevalence Clinical Audit Checklist (2023-2024)**
**Instructions for Nurse-Midwife/ Data Collector:**<br>1. Complete this checklist for **every** mother following delivery and initial postpartum assessment.<br>2. Obtain **oral consent** (Section 1) before proceeding.<br>3. Fill in all applicable fields. Use BLOCK LETTERS.<br>4. File completed checklist securely in the designated study box at the nurse’s station.	

Section 1: Statement of Oral Consent and Data Collector Information

Read the following statement to the mother:
"Hello. As part of a routine health system check to improve services for all women, we are documenting information about mothers' health during childbirth. This includes checking for any past procedures on the female genitalia. Your information will be kept completely confidential and used only to understand community health needs. Your participation is voluntary. Do you agree for me to include your anonymous information in this check?"

Mother Agreement: [ ] YES [ ] NO
(If "NO," thank the mother and STOP here. Do not complete the rest of the form.)

Data Collector's Name: ____ **Signature:**

____________________________ **Date:** //202___

Section 2: Patient General Information for Registry Verification
(To be completed for all consented mothers)

Field	Instruction	Entry
**Facility Code**	*Use your facility's code (see box).*	**[ ] CDH [ ] CHC [ ] MPH [ ] DHC [ ] HHC**
**Date of Delivery**	DD/MM/YYYY	__ / __ / 202__
**Shift at Delivery**	Tick one.	**[ ] Morning [ ] Evening [ ] Night**
**Maternal Age**	In completed years.	__ years
**Sequential Birth Number**	This mother's delivery number for today at this facility (e.g., 1st=1, 2nd=2).	__
**De-identified Mother Code (UID)**	*Create code using formula:* **[Facility Code] - [Last 2 of Year] [Month][Day] - [Seq. Birth No.]** *Example: 5th delivery at CHC on Oct 25, 2023 =* **CHC-231025-05**	________-______-__

Section 3: FGM Specific Clinical Data *(To be completed after physical examination)*

Field	Instruction	Entry
**FGM Status**	Based on visual examination.	**[ ] Positive [ ] Negative**
**If Positive, WHO Type**	*Refer to WHO typology poster.*	**[ ] I [ ] II [ ] III [ ] IV**

Mother's Tribe *As reported by the mother.* ______________________________

INSTRUMENT 3	Interview Guide for FGM-Positive Young Mothers (English Version)	
**Participant Code Number (e.g., KI1, KI2--)**		
**PART A: Participants' Demographics**		
1	Setting (hospital name)	
2	Participant tribe	
3	Participant age (years)	
4	Marital status	
5	Education level	
Qn1	Could you tell me about your own experience with FGM? You might think about when it happened, what you remember, and how you felt at that time.	
Qn2	From your perspective, how is FGM practiced in the Chamwino community today? What, if anything, has changed compared to the past?	
Qn3	In your view, what power do young people or children have to say 'no' to FGM, either for themselves or for their future children? What makes it easy or difficult for them?	
Qn4	Thinking about your life now, in what ways, if any, does having undergone FGM affect your daily life, your health, or your relationships? Could you describe an example of a specific challenge or difficulty you have faced?	
Qn5	Many people in the community say they know FGM is harmful, yet it continues. In your opinion, what are the most important reasons it persists?	
Qn6	What do you see as the most important role for young people, like yourself, in helping the community move away from FGM? What would they need to be able to play that role effectively?	
Qn7	If the community decided to end FGM, what do you think are the most practical and effective steps it could take? Who would need to be involved?	

References

1. World Health Organization. Female genital mutilation. 2025. Available from: https://www.who.int/news-room/fact-sheets/detail/female-genital-mutilation
2. Ministry of Health (MoH) [Tanzania Mainland], Ministry of Health (MoH) [Zanzibar], National Bureau of Statistics (NBS), Office of the Chief Government Statistician (OCGS), and ICF. Tanzania Demographic and Health Survey and Malaria Indicator Survey (TDHSMIS) 2022 Final Report. Dodoma, Tanzania, and Rockville, Maryland, USA: MoH, NBS,
OCGS, and ICF; 2023. Available from:

https://www.nbs.go.tz/uploads/statistics/documents/en-1705490100-Tanzania_DHS-MIS_2022_Final_Report.pdf

3. UNICEF. International Day of Zero Tolerance for Female Genital Mutilation 2023: Partnership with men & boys to transform social and gender norms to end FGM. 2023. Available from: https://www.unicef.org/documents/international-day-zero-tolerancefemale-genital-mutilation-2023
4. UN WOMEN. Ending FGM is essential to give girls control over their own lives. 2021. Available from: https://www.unwomen.org/en/news/stories/2021/2/feature-ending-fgm-isessential
5. Muhula S, Mveyange A, Oti SO, Bande M, Kayiaa H, Leshore C, et al. The impact of community led alternative rite of passage on eradication of female genital mutilation/cutting in Kajiado County, Kenya: A quasi-experimental study. PLoS One. 2021 Apr 1;16(4):e0249662. doi:10.1371/JOURNAL.PONE.0249662 PubMed PMID: 33909635.
6. 28 Too Many. TANZANIA: THE LAW AND FGM. 2018. Available from: https://www.28toomany.org/tanzania/
