# Supplementary material for: Targeting a future generation free from female genital mutilation: A mixed-methods quasi-experimental study of an awareness intervention in central Tanzania
Source: PLOS Glob Public Health. 2026 May 26;6(5):e0006365. doi: 10.1371/journal.pgph.0006365 (PMC13210218; doi:10.1371/journal.pgph.0006365)
Supplement: S1 Table — (PDF) [file pgph.0006365.s005.pdf]

S1 Table. Detailed participant tribes involved in the study baseline (N=468) and endline (N=452), Chamwino District, Tanzania

Manuscript: Targeting a future generation free from female genital mutilation: a mixed-methods quasi-experimental study of an awareness intervention in central Tanzania

Variable	Baseline (N=468)	Endline (N=452)
**Tribe**		
**Bena**	2	2
**Chagga**	10	10
**Digo**	1	1
**Fipa**	5	5
**Gogo**	267	260
**Haya**	7	7
**Hehe**	24	23
**Iraqw**	16	12
**Jita**	3	2
**Kaguru**	9	9
**Kinga**	2	2
**Burunge**	4	4
**Kuria**	6	5
**Luguru**	4	4
**Luo**	1	1
**Maasai**	14	14
**Mang'ati**	1	1
**Manyema**	1	1
**Rangi**	17	16
**Mbugwe**	1	1
**Ngoni**	5	5
**Ngulu**	5	5
**Nyakyusa**	7	7

**Nyamwezi**	5	5
**Nyasa**	1	1
**Nyaturu**	11	10
**Sandawe**	3	3
**Nyiramba**	1	1
**Pare**	7	7
**Sambaa**	2	2
**Sukuma**	19	19
**Zaramo**	1	1
**Zigua**	6	6
**Total tribes (n)**	**33**	**33**
