# Supplementary material for: Targeting a future generation free from female genital mutilation: A mixed-methods quasi-experimental study of an awareness intervention in central Tanzania
Source: PLOS Glob Public Health. 2026 May 26;6(5):e0006365. doi: 10.1371/journal.pgph.0006365 (PMC13210218; doi:10.1371/journal.pgph.0006365)
Supplement: S2 Table — (PDF) [file pgph.0006365.s006.pdf]

S2 Table: Baseline and endline awareness findings stratified by sex characteristics (complete sample, N=452)

Manuscript: Targeting a future generation free from female genital mutilation: a mixed-methods quasi-experimental study of an awareness intervention in central Tanzania

**Variable**		**Baseline (n, %)**			**Endline (n, %)**		
		**Male (n=234)**	**Female (n=234)**	**Total**	**Male (n=228)**	**Female (n=224)**	**Total**
FGM is harmful		211	201	412	223	214	437
Harmful effects of FGM (Multiple responses allowed)	Excessive bleeding	174	159	333	207	193	400
	Severe pain	132	114	246	184	173	357
	Infections	90	72	162	166	145	311
	Urinary problems	71	53	124	125	137	262
	Psychological problems	87	60	147	118	113	231
	Sexual problems	74	63	137	130	122	252
	Difficult childbirth	138	121	259	125	132	257
	Others: long-term scars and structural deformity	1	1	2	0	0	0
FGM is conducted under no medical reasoning		115	122	237	213	206	419
Sources of FGM awareness (Multiple responses allowed)	Learning from school	138	143	281	213	208	421
	Mass media	121	113	234	71	61	132
	Discussions with the community	40	19	59	38	35	73
	Witnessing within the family, relatives, or neighbors	13	17	30	14	14	28
	Positive FGM status (women only)	—	17	17	—	—	—
	Prior involvement in an anti-FGM program	0	0	0	—	—	—
	Others (including hospital education, religious houses, and sexual relationships)	0	3	3	2	5	7
FGM is still occurring in the community		86	93	179	132	98	230
FGM violates the human rights of girls and women		194	193	387	226	215	441
	Intimate partner violence	103	82	185	138	126	264

Forms of violations associated with FGM (Multiple responses allowed)	Child, early, and forced marriage	86	90	176	138	141	279
	Stigma and gender discrimination	82	73	155	136	115	251
	Right to health, security, and physical integrity	70	38	108	131	118	249
	Right to life in cases of death	101	63	164	144	132	276
	Right to be free from torture and cruel, inhuman, or degrading treatment	44	53	97	73	80	153
FGM is criminalized by national and international laws		194	200	394	220	217	437
Knew the 'International Day of Zero Tolerance for FGM, February 6', and its core aim		95	97	192	218	206	424
Desired FGM abandonment		187	180	366	219	214	433
**—Assessed only at baseline; not part of comparative analysis**							
