# Supplementary material for: Targeting a future generation free from female genital mutilation: A mixed-methods quasi-experimental study of an awareness intervention in central Tanzania
Source: PLOS Glob Public Health. 2026 May 26;6(5):e0006365. doi: 10.1371/journal.pgph.0006365 (PMC13210218; doi:10.1371/journal.pgph.0006365)
Supplement: Attachment — Submitted filename: Response to Reviewers.pdf [file pgph.0006365.s008.pdf]

RESPONSE TO REVIEWERS

Manuscript ID: PGPH-D-25-02903R1

Title: Targeting a future generation free from female genital mutilation: a mixed-methods quasi-experimental study of an awareness intervention in central Tanzania

Date: March 30, 2026.

To the Editor and Reviewers,

We thank you for the opportunity to revise our manuscript and for the reviewers' dedicated time and constructive comments. Their insights have been invaluable in strengthening the methodological clarity, statistical reporting, and overall presentation of our work. We also appreciate the editor's detailed guidance on the quantitative analysis.

We would like to extend our sincere gratitude to Reviewer #1, who reviewed the initial version of this manuscript and confirmed that all previous comments were adequately addressed. We appreciate their time and positive assessment, which encouraged us to continue refining the work. We are also grateful to the editor for facilitating the review process and to Reviewer #2 for providing additional thoughtful feedback that has further strengthened the manuscript.

We have carefully addressed every point raised. The key improvements include:

1. Reconceptualized the primary outcome as the continuous mean awareness score, removing arbitrary categorization and replacing modified Poisson and logistic regression with linear regression, consistent with the editor's guidance.

2. Clarified the 19-point awareness scale, detailing its composition and scoring, and added a justification for excluded items based on the distinction between factual awareness and social norms [26].

3. Added baseline findings in a combined Table 4, addressing the request for baseline data, and all generated data during the analysis of baseline and endline findings have been included in the manuscript sections and their supporting files.

4. Integrated key references (Matanda et al., 2023 [4] and WHO Guideline, 2025 [6]) throughout the Introduction, Methods, and Discussion.

5. Defined fidelity of implementation with three specific criteria in the Results section.

6. Provided a reflection on the higher FGM prevalence among young mothers (31.0%) in the Discussion, with explanations including cohort effects, global evidence, and underreporting.

7. Discussed the feasibility of the 19-point scale and offered recommendations for future adaptations.

8. Added a new section on costs and scalability in the Implications, describing how the intervention leverages existing structures and could be integrated into domestically funded services.

Below, we provide a point-by-point response to each comment.

**Part A: Addressing Editor's Comments**			
**Area**	**Editor Comment**	**Author Response**	**Location in Manuscript**
**1. Awareness Scale Clarification**	You describe a 19-item knowledge scale, but the data collection instrument detailed in the S3 file contains 12 questions on Knowledge, Attitude, and Perceptions, some of which are noted as excluded from the awareness level estimation. It is not clear	We thank the editor for this important clarification. The instrument contained 12 questions with sub-items, yielding a total possible score of 19 points (1 point per correct response). To avoid confusion, we now refer to a "19-point	Methods – Outcomes and measurement (page 11-13); S4 File (Data Collection Instruments)

	what the 19 items in the knowledge scale are. Please clarify what the 19 items are, and how each was scored. Please also consider consistency in terminology: is this measure Knowledge or Awareness?	composite score" rather than a "19-item scale." The scoring method is explicitly described in the Methods section, and the supplementary materials (S4 File) have been updated with a detailed scoring key. We have consistently used the term "awareness" throughout to maintain terminological clarity.	
**2. Inclusion of Baseline Results**	Please provide baseline results as well as the endline results reported in Table 4.	We have revised Table 4 to present both baseline and endline results side by side, with p-values from McNemar's test or paired t-test as appropriate. The table now includes all key awareness items at both time points. Additionally, all table results now consolidate both time points' results and the S2 Table. Baseline and endline awareness findings, stratified by sex characteristics (complete sample, N=452), are provided in a supplementary file.	Results – Table 4 (page 21); all table results and S2 Table.

**3. Categorization of Continuous Data**	The primary outcome is described as "the change in the proportion of participants with 'Adequate Awareness' of FGM health risks, defined as a score >76% on a 19-item knowledge scale, with scores categorized as inadequate (<50%), moderate (50–75%), and adequate (>76%)"<br><br>It is not at all clear what the basis for these categories is, or why the scores even should be categorized. You cite Kafle et al. (2020), but they provide no rationale or justification for the categorization of continuous data. Unless there is a statistical/distributional reason (e.g., bimodal distribution of data), or a well-defined a priori cutoff (e.g., a physiological parameter that falls above/below a critical threshold), continuous data should not be divided into arbitrary categories.<br><br>Therefore, please could you treat your	We agree with the editor's methodological guidance. We have removed the arbitrary categorization (inadequate, moderate, adequate) from the primary analysis. The primary outcome is now the change in the mean awareness score (continuous), analyzed using a paired t-test, with Cohen's d as the effect size. For demographic associations, we now use linear regression, reporting unstandardized beta coefficients (B) with 95% confidence intervals. The categorization is retained only as descriptive context in Table 4, with a footnote clarifying that the primary analysis used the continuous score.	Methods – Data analysis (page 14-15); Results – Changes in FGM awareness score (baseline–endline) (page 19); Table 4 (page 21)

	knowledge/awareness data as continuous in all analyses, which means replacing the modified Poisson regression and logistic regression with linear regression analyses.<br>Could you please carefully revise the manuscript to address all comments raised?		
**Part B: Addressing Reviewer 2 Comments**			
**Area**	**Editor Comment**	**Author Response**	**Location in Manuscript**
**1. Missing Intervention References**	The evidence on what works - systematic review of interventions of seminal publications references below) such are NOT referenced or referred to in the intervention design rationale or discussion. a) Matanda DJ, et al. What interventions are effective to prevent or respond to female genital mutilation? A review of existing evidence from 2008–2020. PLOS Glob Public Heal. b) World Health Organization. (2025). WHO guideline on the prevention of female genital mutilation and	We thank the reviewer for highlighting these important references. We have now integrated both throughout the manuscript. Matanda et al. [4] is cited in the Introduction to highlight the evidence gap on community interventions that shift attitudes without behaviour change, and in the Discussion to contextualize our "awareness-power gap" finding. The WHO guideline [6] is cited in the Introduction as global guidance for educational interventions, in the Methods to support nurse-	Introduction (page 3-4); Methods – Interventions (page 10-11); Discussion (pages 28-33)

	clinical management of complications.	midwife training, and in the Discussion to align our findings with WHO recommendations.	
**2. Intervention Duration**	The intervention duration is not clear. How long was the exposure?	We have clarified that the intervention was delivered over a 6-month period (September 2023 – February 2024), with the overall study spanning 12 months from baseline to endline.	**Methods – Interventions** (page 10-11), first paragraph
**3. TDHS-MIS Baseline and Comparison**	The authors should explain why 2022 TDHS-MIS data on FGM prevalence/knowledge in Dodoma was not considered for baseline and also compare the survey findings on FGM prevalence, knowledge and attitudes with this study results.	We have addressed this in two locations. First, in the Outcomes section, we explain that TDHS-MIS data were not used as a baseline comparator because they are aggregated at the regional level, whereas our study focused specifically on the Chamwino District. Second, we added a new subsection	Methods – Outcomes and measurement (page 11-13); Discussion – Comparison with national and regional data (page 28)

		in the Discussion ("Comparison with national and regional data") that compares our findings on prevalence, attitudes, and underreporting with TDHS-MIS estimates.	
**4. Rationale for Knowledge as Success Measure**	The authors would need to explain why they chose "adequacy of knowledge of health risks" as success based on existing literature which does not support the rationale that "knowledge of health risks" is associated with behavioral/attitudinal or behavior change specifically on FGM.	We have clarified that knowledge/awareness is a necessary precursor to behaviour change, though not sufficient on its own, a finding our qualitative data reinforces. In the Introduction, we state our hypothesis that awareness will increase. In the Discussion, we clearly note that while awareness improved, it did not reliably translate into abandonment intention, consistent with Matanda et al. [4]. This	Introduction (page 3-4), final paragraph; Discussion – Awareness-power gap (page 30)

		framing acknowledges the limitations of knowledge-focused interventions while justifying our focus on this intermediate outcome.	
**5. Definition of Fidelity**	What was the definition of strong fidelity of implementation? Can this be elaborated?	We have added a clear definition in the Results section. Fidelity was assessed through three criteria: (1) adherence to the validated training manual across all sessions; (2) delivery of core educational content by trained champions; and (3) consistent use of standardized materials as verified through field observations and champion reports.	Results – Intervention reach, fidelity, and process evaluation (page 16)
6. Higher Prevalence	The higher prevalence of FGM among young mothers (15-19) - almost double of	We have added a reflection in the Discussion. We explain that the	Discussion – Comparison with national and regional data (page 28)

Among Young Mothers	overall FGM prevalence warrants an explanation/reflections.	disproportionate burden among young mothers (31.0%) may reflect a cohort effect (cut more recently during the shift to early childhood secrecy), aligns with global evidence of age-cohort variation in FGM prevalence, and highlights underreporting driven by fear of legal consequences and social desirability bias.	
**7. Feasibility of 19-Point Scale**	From a practicality point of view - is a 19 scale knowledge scale feasible to be conducted? Are the authors reconsidering or providing a recommendation given the findings and other systematic reviews whether this tool was adequate or requires revision?	We have added a paragraph in the Strengths, limitations section addressing feasibility and recommendations. The scale was feasible (10–12 minutes completion time, 96.6% retention, Cronbach's $\alpha = 0.70$). Based on our findings and Matanda et al.	Discussion – Strengths, limitations, and generalizability (page 32)

		[4], we recommend future adaptations: (1) shortening the scale while preserving core items; (2) adding items probing reasons for FGM persistence directly; and (3) integrating qualitative sub-questions to contextualize quantitative responses.	
**8. Costs and Scalability**	Implications for policy or strategy, one key area is costs and scalability of this intervention. If the intervention is dependent on siloed approach dependent on external funds, chances that it is not scalable/affordable. Authors would need to speak to how this intervention could be part of existent domestically funded services that have a wide reach.	We have added a new point (point 4) in the Implications section. The intervention leveraged existing community structures (schools, households, health facilities, local leaders), reducing the need for parallel infrastructure. Champions were volunteers, and activities were integrated into routine health and education settings. Rather than relying on external funding, this model aligns with existing domestically funded services, suggesting scalability within school	Discussion – Implications for policy and practice (point 4, page 32)

		health programs, reproductive health services, and community health worker networks. We acknowledge the need for formal cost-effectiveness analysis.	

We believe these revisions have substantially improved the manuscript. The study design is now clearly defined as a mixed-methods quasi-experimental study with a continuous primary outcome analyzed using appropriate parametric methods. The measurement tool is transparently described, and the analytical approach is consistent with best practices for reporting continuous outcomes. All reviewer comments have been addressed, and we have added new content on fidelity, feasibility, young mothers' prevalence, costs and scalability, and integrated key references.

We thank the editor and reviewers for their thoughtful feedback, which has helped us produce a stronger manuscript. We look forward to your decision.

Sincerely,

Leah Barthalome Kimario

Corresponding author

On behalf of all co-authors
