# Supplementary material for: Targeting a future generation free from female genital mutilation: A mixed-methods quasi-experimental study of an awareness intervention in central Tanzania
Source: PLOS Glob Public Health. 2026 May 26;6(5):e0006365. doi: 10.1371/journal.pgph.0006365 (PMC13210218; doi:10.1371/journal.pgph.0006365)
Supplement: Attachment — Submitted filename: Response to Reviewers.docx [file pgph.0006365.s007.docx]

**RESPONSE TO REVIEWERS**

**To the Editor and Reviewers of PLOS Global Public Health,**

We thank you for the opportunity to revise our manuscript and for the reviewers’ dedicated time and constructive comments. Your insights have been invaluable in strengthening the methodological clarity, statistical reporting, and overall presentation of our work, “Targeting a future generation free from female genital mutilation: a mixed-methods quasi-experimental study of an awareness intervention in central Tanzania.”

We have carefully addressed every point raised. The key improvements include:

1. **Correcting the study design terminology** from “cross-sectional” to “mixed-methods, single-arm, pre-post quasi-experimental study” throughout.
2. **Justifying all statistical tests and reporting effect sizes with confidence intervals** alongside p-values.
3. **Streamlining the manuscript** by reducing repetition in the Introduction, Methods, and Discussion.
4. **Adding a participant flow diagram** (CONSORT-style) to clarify the sampling process.
5. **Explicitly discussing the study’s limitations,** including the lack of a control group and risk of contamination, with greater depth.

Below is a point-by-point response detailing our actions. All changes are integrated into the revised manuscript file, and a version with tracked changes is provided for your convenience.

| **Area** | **Reviewer Comment** | **Author Response** | **Location in Manuscript** |
| --- | --- | --- | --- |
| **1. Clarify Study Design** | The manuscript calls itself "mixed-methods cross-sectional," yet includes pre-post data and longitudinal follow-up. This is not cross-sectional. Discuss and cite <https://pubmed.ncbi.nlm>.  nih.gov/37539674/. Clarify the design: this appears to be quasi-experimental pre-post intervention with explanatory qualitative component. | We thank the reviewer for this critical correction. The manuscript no longer uses the term “cross-sectional” or “observational study.” We have clearly defined the design as a **“community-based, mixed-methods study"** with a primary **"single-arm, baseline-endline quasi-experimental design (non-randomized)"** and a concurrent qualitative component. This precise terminology is now used consistently from the title through the methods. Regarding the suggested citation (PMID: 37539674), we reviewed the article, which is a valuable narrative review of FGM complications. While it does not directly discuss study design terminology, we agree with its emphasis on the severe and under-investigated consequences of FGM, a central motivation for our intervention. We have therefore cited it appropriately in the introduction to highlight the health rationale for our study. | **Title, Abstract, “Study design and registration” section (Page 1-4).** The reference is now cited in the Introduction (Page 3). |
| **2. Simplify Sampling and Provide Flow Chart** | The sampling approach is confusing; simplify and provide a CONSORT-style flow chart. | We have simplified the sampling description in the text. Furthermore, as requested, we have created and included a **CONSORT-style participant flow diagram** (Fig 1) that visually summarizes the multi-stage sampling, enrollment, and follow-up for all study components (quantitative pre-post, qualitative interviews, prevalence audit). | **“Participants’ sampling, recruitment, and eligibility criteria” section (Page 6-8) and new Fig 1 (Page 8)/S1_Fig (separate file).** |
| **3. Justify Statistical Tests** | Some tests are not justified (e.g., paired t-test for awareness scores—were these normally distributed?). | We have added justification for the paired t-test. The analysis now states: **“The primary outcome... was assessed using a paired t-test after confirming normality (Shapiro-Wilk test: W = .995, p = .130).”** The suitability of other tests (McNemar’s, Chi-square, regression) for their respective outcome types is also clearly stated. | **“Data analysis” section, paragraph 1 (Page 11-12).** |
| **4. Rationale for Prevalence Ratios** | Provide rationale for using prevalence ratios rather than risk ratios. | We have added the rationale for using Adjusted Prevalence Ratios (aPR). The text now explains: **“Intervention effect sizes were estimated using aPRs with 95% CIs via Modified Poisson Regression with robust error variances. This method provides direct, interpretable estimates of effect for common categorical outcomes and is more appropriate than odds ratios when outcomes are not rare.”** | **“Data analysis” section, paragraph 2 (Page 12).** |
| **5. Report Effect Sizes and Simplify Analysis** | Many p-values reported without effect sizes in text. Action: (1) State test assumptions and verify data suitability (2) Report effect sizes and confidence intervals consistently, (3) Consider simplifying analytic presentation for readability. | We have comprehensively addressed this. (1) Test assumptions are stated (see response to #3). (2) **Effect sizes and 95% Confidence Intervals are now reported consistently for all key results** (e.g., mean difference, aPR, aOR). P-values are provided alongside them. (3) The presentation has been streamlined by consolidating repetitive analytical descriptions and using clear, consistent language for each statistical test applied. | **"Data analysis" section and all results subsections (e.g., "Intervention impact...", "Changes in attitudes...") (Pages 11-16).** |
| **6. No Control Group Limitation** | No true control group; intervention effect cannot be isolated from secular trends or social desirability bias. | We fully acknowledge this important limitation. The Discussion now contains an expanded paragraph that explicitly states: **"The main limitation is the baseline-endline, single-arm design, which cannot definitively establish causality; observed changes might be influenced by secular trends, increased community discussion, or social desirability bias."** We discuss the implications of this for interpreting the findings. | **"Strengths and limitations" section, (Page 26).** |
| **7. Risk of Contamination** | Significant risk of contamination (e.g., community discussion and school-based diffusion). | We have integrated this point into the limitation on the lack of a control group. The revised text notes that the community-based nature of the intervention means **"educational diffusion beyond direct participants was possible,"** which, while a potential threat to internal validity, could also be viewed as a positive real-world effect of the campaign. We frame this as an inherent challenge of community participatory research. | **"Strengths and limitations" section, (Page 26).** |
| **8.-11. Streamline Manuscript** | Manuscript is lengthy and repetitive; consider streamlining:  Introduction too long—reduce general material, increase Tanzania-specific gaps  Methods repetitive in intervention description  Discussion reiterates results instead of synthesizing | We have significantly condensed the manuscript to improve readability:  **Introduction:** Reduced general background on FGM, focusing more on the **specific gap in Tanzania and the Dodoma/Chamwino context.**  **Methods:** Removed repetitive descriptions of the intervention across sub-sections, consolidating details into a single, clear **"Interventions"** paragraph.  **Results:** Streamlined the entire section including presenting the qualitative findings in connected explanation of the participants responses and direct quotes, to remove redundancy.  **Discussion:** Restructured to **synthesize findings and compare with literature** rather than re-stating results. The "Implications for policy" section now directly flows from the novel synthesis of quantitative and qualitative data (the "knowledge-power gap"). |  |
